# The Asian Pacific Association of the Study of the Liver expert survey on artificial intelligence-assisted reporting of liver histopathology in metabolic dysfunction associated fatty liver disease

**DOI:** 10.1007/s12072-026-11092-6

**Published:** 2026-05-13

**Authors:** H. Elangovan, K. Akbary, A. Rastogi, A. Wee, G. Soon, L. Adams, E. Carr-Boyd, A. Clouston, C. L. Cooper, W. K. Chan, Y. Y. Dan, R. Dela-Cruz, G. Goh, S. S. Hamid, D. Q. Huang, T. Kawaguchi, H. Kim, W. Kim, S. U. Kim, J. D. Jia, C. J. Liu, F. Liu, W. Q. Leow, M. D. Muthiah, I. Ng, D. Payawal, A. F. Pan, S. Pervez, G. Shiha, T. Tanwandee, Y. Tanaka, L. Thiyaphat, M. Vij, Y. Yilmaz, F. Yilmaz, M. L. Yu, K. Zalata, M. H. Zheng, J. G. Fan, S. K. Sarin, V. Wong, J. George

**Affiliations:** 1 Storr Liver Centre, The Westmead Institute for Medical Research, Westmead Hospital, The University of Sydney, Sydney, NSW Australia; 2 HistoIndex Pte Ltd, Singapore, Singapore; 3 Department of Pathology, Institute of Liver and Biliary Sciences, New Delhi, India; 4 Department of Pathology, National University Hospital, Singapore, Singapore; 5 Department of Anatomical Pathology, Singapore General Hospital, Singapore, Singapore; 6 Medical School, University of Western Australia, Perth, Washington, Australia; 7 Histopathology Department, Auckland City Hospital, Auckland, New Zealand; 8 Centre for Liver Disease Research, School of Medicine (Southern), University of Queensland, Princess Alexandra Hospital, Ipswich, Australia; 9 Pathology Queensland, Princess Alexandra Hospital, Woolloongabba, Qld Australia; 10 Gastroenterology and Hepatology Unit, Department of Medicine, Faculty of Medicine, University of Malaya, Kuala Lumpur, Malaysia; 11 Division of Gastroenterology and Hepatology, Department of Medicine, National University Hospital, Singapore, Singapore; 12 The Medical City, Ortigas, Pasig City, Metro Manila, Philippines; 13 Department of Gastroenterology and Hepatology, Singapore General Hospital, Singapore, Singapore; 14 Department of Medicine, Aga Khan University, Karachi, Pakistan; 15 Division of Gastroenterology, Department of Medicine, Kurume University School of Medicine, Kurume, Japan; 16 Department of Pathology, Seoul National University Hospital, Seoul National University College of Medicine, Seoul, Republic of Korea; 17 Division of Gastroenterology and Hepatology, Department of Internal Medicine, Seoul Metropolitan Government Boramae Medical Center, Seoul National University College of Medicine, Seoul, Republic of Korea; 18 Department of Internal Medicine, Yonsei University College of Medicine, Seoul, South Korea; 19 Liver Research Center, Beijing Friendship Hospital, Capital Medical University, Beijing, 100050 China; 20 Division of Gastroenterology and Hepatology, Department of Internal Medicine, National Taiwan University Hospital, Taipei, Taiwan; 21 Peking University People’s Hospital, Peking University Hepatology Institute, Infectious Disease and Hepatology Center, Peking University People’s Hospital, Beijing, China; 22 Department of Pathology and State Key Laboratory of Liver Research, The University of Hong Kong, Pok Fu Lam, Hong Kong; 23 Department of Internal Medicine, Fatima University Medical Center, Valenzuela, Philippines; 24 Department of Pathology, Queen Elizabeth Hospital, Kota Kinabalu, Sabah, Malaysia; 25 Department of Pathology, Aga Khan University, Karachi, Pakistan; 26 Gastroenterology and Hepatology Unit, Internal Medicine Department, Faculty of Medicine, Mansoura University, Mansoura, Egypt; 27 Division of Gastroenterology, Department of Medicine, Faculty of Medicine Siriraj Hospital, Bangkok, Thailand; 28 Department of Gastroenterology and Hepatology, Kumamoto University, Kumamoto, Japan; 29 Division of Pathology, Chulabhorn International College of Medicine, Thammasat University, Pathum Thani, Thailand; 30 Department of Pathology, Dr Rela Institute and Medical Center, Chennai, Tamil Nadu India; 31 Department of Gastroenterology, School of Medicine, Recep Tayyip Erdoğan University, Rize, Türkiye; 32 Faculty of Medicine, Department of Pathology, Ege University, Izmir, Türkiye; 33 Hepatobiliary Division, Department of Internal Medicine, Center of Hepatitis Research, College of Medicine and Center of Metabolic Disorders and Obesity, Kaohsiung Medical University Hospital, Kaohsiung Medical University, Kaohsiung, Taiwan; 34 Anatomic Pathology Department, Faculty of Medicine, Mansoura University, Mansoura, Egypt; 35 Department of Hepatology, MAFLD Research Center, The First Affiliated Hospital of Wenzhou Medical University, Wenzhou, China; 36 Center for Fatty Liver, Department of Gastroenterology, Shanghai Key Lab of Pediatric Gastroenterology and Nutrition, Xinhua Hospital Affiliated to Shanghai Jiao Tong University School of Medicine, Shanghai, 200092 China; 37 Department of Hepatology, Institute of Liver and Biliary Sciences, New Delhi, India; 38 Department of Medicine and Therapeutics, Faculty of Medicine, The Chinese University of Hong Kong, Sha Tin, Hong Kong

**Keywords:** MAFLD, MASH, Artificial Intelligence, Histopathology

## Abstract

**Introduction:**

Artificial intelligence (AI) and digital pathology have the potential to augment liver biopsy interpretation in MAFLD in clinical practice and trials assessment. However, attitudes and barriers to its implementation have not been systematically explored.

**Methods:**

A survey focusing on conventional liver histology, digital pathology and its AI applications in MAFLD/MASH was conducted among hepatologists and liver pathologists in the Asia Pacific region.

**Results:**

AI-assisted digital pathology is perceived to be a valuable addition to existing histological reporting in MAFLD/MASH. Defined standards for application and validation of AI models are important priorities for their implementation.

**Conclusion:**

There is consensus among clinical experts in the Asia Pacific that AI-assisted histological assessment is useful in MAFLD/MASH interpretation. However, there remain important challenges to the adoption of these technologies into routine clinical workflows.

**Supplementary Information:**

The online version contains supplementary material available at 10.1007/s12072-026-11092-6.

## Introduction

Histopathological interpretation of liver biopsies represents the current gold standard for the assessment of treatment effect in drug trials of metabolic dysfunction associated fatty liver disease (MAFLD), and its progressive subtype metabolic dysfunction associated steatohepatitis (MASH) [1]. Advancements in digital pathology provide opportunities to enhance biopsy interpretation through artificial intelligence (AI) augmentation to gain nuanced insights into tissue architecture. Moreover, tools such as second harmonic generation/two photon excitation fluorescence (SHG/TPEF) microscopy to assess collagen morphology in unstained liver biopsies avoid quality control concerns from traditional stained slide workflows and allow for developing continuously scaled histologic scoring metrics. These innovations may drive more sensitive and novel insights to refine trial recruitment, determine treatment efficacy[2] and predict clinical outcomes prediction [3].

As an epicenter of MAFLD with prevalence estimates of 40% and harboring 80% of the global MASH burden [4], the Asia Pacific region is positioned to benefit from the technical enhancements that AI-assisted digital pathology can accord to current histological diagnostics. We report the results of an expert survey to assess the opinions of hepatologists and clinical pathologists in this region with respect to these new technologies in the MAFLD domain.

## Methods

This questionnaire on current attitudes towards liver biopsy assessment, digital pathology and AI applications in MAFLD/MASH was conducted among selected expert hepatologists and liver pathologists in the Asia Pacific region.

The survey was developed following an expert meeting convened at the 2025 Asia Pacific Association for the Study of the Liver (APASL) Congress in Beijing, China. At this meeting, the rationale for this survey, gauging the interest of the expert group, and selecting experts to lead development of the survey were undertaken. This meeting was chaired by JG, SS and JF. Afterwards, emails were sent to 26 hepatologists and 19 clinical pathologists across 15 countries representing the Asia–Pacific region. Of the invitees, 23 hepatologists and 13 pathologists agreed to participate.

The survey was divided into two arms, one for hepatologists, and another focused on clinical pathologists to more accurately capture their respective real-world perspectives on liver biopsy interpretation in clinical practice. The survey questions were conceptualized over three iterations by two hepatologists (JG and VW) and three expert liver pathologists (AR, AW, GS). These clinicians did not participate in the final survey.

The questionnaire was divided into four categories. The first section requested basic demographic information. The second part looked at opinions around conventional liver biopsy. The third section assessed attitudes toward the role of digital pathology and AI in augmenting liver biopsy interpretation in MAFLD/MASH. The final part focused on understanding opinions around quality control related to the adoption of AI and digital pathology in clinical workflows. This section included additional questions for pathologists elucidating their thoughts on current ordinal MAFLD/MASH reporting standards, the interpretability and accuracy of digital pathology images in capturing key histopathological features of MAFLD, accessibility and knowledge of SHG/TPEF microscopy and quantitative fibrosis assessments, and barriers to implementation in the Asia Pacific region. The finalized full question set can be viewed in the accompanying Supplement 1.

Responses were recorded on a Likert scale with the following options: *strongly agree, agree, neither agree nor disagree, disagree, strongly disagree,* and *do not know/prefer not to answer.* For analysis, “overall agree” was defined as the combined proportion of *agree* and *strongly agree* responses, while “overall disagree” comprised *disagree* and *strongly disagree* responses. These aggregated categories were used to facilitate the interpretation of respondent preferences.

### Data analysis

The survey responses were analyzed and the categorical data are presented as percentages (N [%]) of total respondents. The level of agreement required for consensus was defined at 66%.

### Ethics

This survey was conducted in accordance with the European Pharmaceutical Market Research Association (EphMRA) code of ethics. As it is not a clinical study, ethics committee review and approval were not required.

## Results

Key results of the survey are highlighted in this manuscript. The full cumulated responses can be compared in Supplement 2 and individual de-identified responses can be reviewed in Supplement 3, respectively.

## 23 clinical hepatologists and 13 pathologists (*n* = 36) out of 45 invitees participated in the survey. 96% (*n* = 22) of hepatologists and 77% (*n* = 10) pathologists had > 11 years of experience in liver pathology.

### Hepatologist responses

Most hepatologists agreed that conventional liver histology is an imperfect tool to evaluate MAFLD/MASH and to benchmark against non-invasive testing strategies (NITs) (Table 1).

**Table 1:** Hepatologists’ opinions on the performance of conventional liver histology in evaluating MASH/MAFLD

	**Strongly Agree**	**Agree**	**Overall Agree**	**Neither Agree nor Disagree**	**Disagree**	**Strongly Disagree**	**Overall Disagree**	**Do not know/prefer not to answer**
Liver histology is an imperfect gold standard for the evaluation of MASH and for the grading and staging of histological features of MAFLD	10 (43.48%)	10 (43.48%)	20 (86.96%)	0 (0%)	2 (8.7%)	1 (4.35%)	3 (13.04%)	0 (0%)
Liver histology is an imperfect gold standard for the development of non-invasive tests for MASH diagnosis and prognosis	9 (39.13%)	11 (47.83%)	20 (86.96%)	0 (0%)	3 (13.04%)	0 (0%)	3 (13.04%)	0 (0%)
Restricted ordinal histological categories of fibrosis staging are suboptimal for assessing fibrosis progression/regression in clinical trials	12 (52.17%)	9 (39.13%)	21 (91.3%)	1 (4.35%)	0 (0%)	1 (4.35%)	1 (4.35%)	0 (0%)

There was consensus among hepatologists on the need to incorporate digital pathology and AI with existing histological appraisal to enhance MASH interpretation (Table 2).

**Table 2:** Hepatologists’ opinions on the need to incorporate digital pathology and AI interpretation in MASH/MAFLD

	**Strongly Agree**	**Agree**	**Overall Agree**	**Neither Agree nor Disagree**	**Disagree**	**Strongly Disagree**	**Overall Disagree**	**Do not know/prefer not to answer**
There is a need to integrate Digital Pathology with image analysis and/or artificial intelligence-based analysis with existing histological evaluation for fibrosis staging in the evaluation of MASH	14 (60.87%)	8 (34.78%)	22 (95.65%)	0 (0%)	0 (0%)	0 (0%)	0 (0%)	1 (4.35%)
There is a need to integrate Digital Pathology with image analysis and/or artificial intelligence-based analysis with existing histological evaluation for grading of steatosis in the evaluation of MASH	12 (52.17%)	7 (30.43%)	19 (82.61%)	1 (4.35%)	2 (8.7%)	0 (0%)	2 (8.7%)	1 (4.35%)
There is a need to integrate Digital Pathology with image analysis and/or artificial intelligence-based analysis with existing histological evaluation for grading of ballooning in the evaluation of MASH	14 (60.87%)	8 (34.78%)	22 (95.65%)	0 (0%)	0 (0%)	0 (0%)	0 (0%)	1 (4.35%)
There is a need to integrate Digital Pathology with image analysis and/or artificial intelligence-based analysis with existing histological evaluation for grading of lobular inflammation in the evaluation of MASH	12 (52.17%)	10 (43.48%)	22 (95.65%)	0 (0%)	0 (0%)	0 (0%)	0 (0%)	1 (4.35%)

Most surveyed hepatologists believed that digital pathology and AI-assisted image analysis can improve histological assessment standards in MAFLD/MASH (Table 3).

**Table 3:** Hepatologists’ opinions on the utility of digital pathology and AI in MASH/MAFLD

	**Strongly Agree**	**Agree**	**Overall Agree**	**Neither Agree nor Disagree**	**Disagree**	**Strongly Disagree**	**Overall Disagree**	**Do not know/prefer not to answer**
DP/AI can improve the reproducibility of fibrosis scoring in MASH	14 (60.87%)	8 (34.78%)	22 (95.65%)	0 (0%)	0 (0%)	0 (0%)	0 (0%)	1 (4.35%)
DP/AI can improve the reproducibility of steatosis, lobular inflammation, and ballooning scoring in MASH	12 (52.17%)	9 (39.13%)	21 (91.3%)	1 (4.35%)	0 (0%)	0 (0%)	0 (0%)	1 (4.35%)
DP/AI can reduce subjectivity in interpreting borderline MASH grading and fibrosis stages	10 (43.48%)	12 (52.17%)	22 (95.65%)	0 (0%)	0 (0%)	0 (0%)	0 (0%)	1 (4.35%)
DP/AI can be an effective decision support tool for pathologists in grading and staging MASH	8 (34.78%)	14 (60.87%)	22 (95.65%)	0 (0%)	0 (0%)	0 (0%)	0 (0%)	1 (4.35%)
DP/AI should supplement, not replace, human pathology review in clinical practice	11 (47.83%)	9 (39.13%)	20 (86.96%)	3 (13.04%)	0 (0%)	0 (0%)	0 (0%)	0 (0%)
DP/AI based fibrosis evaluation can serve as the comparator for development of non-invasive tests	8 (34.78%)	11 (47.83%)	19 (82.61%)	2 (8.7%)	1 (4.35%)	0 (0%)	1 (4.35%)	1 (4.35%)
Development, validation and standardization of DP/AI tools should be an area of high priority in assessing MAFLD/MASH, particularly for clinical trials and drug development	12 (52.17%)	11 (47.83%)	23 (100%)	0 (0%)	0 (0%)	0 (0%)	0 (0%)	0 (0%)

### Pathologist responses

Contrary to hepatologists’ opinion, pathologists demonstrated a greater degree of disagreement or ambivalence on the perceived inadequacies of conventional liver histological assessment in evaluating MAFLD/MASH and in calibrating NITs (Table 4).

**Table 4:** Liver pathologists’ opinions on the performance of conventional liver histology in evaluating MASH/MAFLD

	**Strongly Agree**	**Agree**	**Overall Agree**	**Neither Agree nor Disagree**	**Disagree**	**Strongly Disagree**	**Overall Disagree**	**Do not know/prefer not to answer**
Liver histology is an imperfect gold standard for the evaluation of MASH and for the grading and staging of histological features of MAFLD	3 (23.08%)	5 (38.46%)	8 (61.54%)	1 (7.69%)	4 (30.77%)	0 (0%)	4 (30.77%)	0 (0%)
Liver histology is an imperfect gold standard for the development of non-invasive tests for MASH diagnosis and prognosis	3 (23.08%)	3 (23.08%)	6 (46.15%)	1 (7.69%)	4 (30.77%)	1 (7.69%)	5 (38.46%)	1 (7.69%)
Restricted ordinal histological categories of fibrosis staging are suboptimal for assessing fibrosis progression/regression in clinical trials	0 (0%)	4 (30.77%)	4 (30.77%)	5 (38.46%)	4 (30.77%)	0 (0%)	4 (30.77%)	0 (0%)

Nonetheless, there was strong support among pathologists for the role of digital pathology and AI-assisted image analysis in augmenting histological assessment standards in MAFLD/MASH (Table 5).

**Table 5:** Liver pathologists’ opinions on the utility of digital pathology and AI in MASH/MAFLD

	**Strongly Agree**	**Agree**	**Overall Agree**	**Neither Agree nor Disagree**	**Disagree**	**Strongly Disagree**	**Overall Disagree**	**Do not know/prefer not to answer**
DP/AI can improve the reproducibility of fibrosis scoring in MASH	3 (23.08%)	8 (61.54%)	11 (84.62%)	2 (15.38%)	0 (0%)	0 (0%)	0 (0%)	0 (0%)
DP/AI can improve the reproducibility of steatosis, lobular inflammation, and ballooning scoring in MASH	2 (15.38%)	8 (61.54%)	10 (76.92%)	1 (7.69%)	2 (15.38%)	0 (0%)	2 (15.38%)	0 (0%)
DP/AI can reduce subjectivity in interpreting borderline MASH grading and fibrosis stages	2 (15.38%)	9 (69.23%)	11 (84.62%)	0 (0%)	2 (15.38%)	0 (0%)	2 (15.38%)	0 (0%)
DP/AI can be an effective decision support tool for pathologists in grading and staging MASH	2 (15.38%)	10 (76.92%)	12 (92.31%)	1 (7.69%)	0 (0%)	0 (0%)	0 (0%)	0 (0%)
DP/AI should supplement, not replace, human pathology review in clinical practice	8 (61.54%)	4 (30.77%)	12 (92.31%)	0 (0%)	0 (0%)	1 (7.69%)	1 (7.69%)	0 (0%)
DP/AI based fibrosis evaluation can serve as the comparator for development of non-invasive tests	2 (15.38%)	9 (69.23%)	11 (84.62%)	2 (15.38%)	0 (0%)	0 (0%)	0 (0%)	0 (0%)
Development, validation and standardization of DP/AI tools should be an area of high priority in assessing MAFLD/MASH, particularly for clinical trials and drug development	3 (23.08%)	9 (69.23%)	12 (92.31%)	1 (7.69%)	0 (0%)	0 (0%)	0 (0%)	0 (0%)

5 of the 13 surveyed pathologists had previously utilized digital pathology or AI applications in liver histological assessment and only 1 pathologist had operational experience with SHG/TPEF microscopy. Consequently, most respondents reported unfamiliarity with the capabilities of SHG/TPEF microscopy over conventional fibrosis staging. 9 out of 13 pathologists agreed that AI models that provide continuous metrics in histological interpretation will be helpful to pathology practice (Table 6).

**Table 6:** Liver pathologists’ opinions on SHG/TPEF-based microscopy to evaluate MASH/MAFLD

	**Strongly Agree**	**Agree**	**Overall Agree**	**Neither Agree nor Disagree**	**Disagree**	**Strongly Disagree**	**Overall Disagree**	**Do not know/prefer not to answer**
DP/AI on unstained sections, including Second Harmonic Generation (SHG)-based imaging, is advantageous to DP/AI on stained sections due to circumvention of potential pre-analytical errors (e.g., staining variability)	0 (0%)	1 (7.69%)	1 (7.69%)	5 (38.46%)	0 (0%)	0 (0%)	0 (0%)	7 (53.85%)
SHG-based images have better resolution and contrast than routine histochemical stains for fibrosis assessment	0 (0%)	2 (15.38%)	2 (15.38%)	3 (23.08%)	0 (0%)	0 (0%)	0 (0%)	8 (61.54%)
SHG-based images alone without AI solutions can assist pathologists in MASH biopsy evaluation, especially for fibrosis assessment	0 (0%)	2 (15.38%)	2 (15.38%)	1 (7.69%)	2 (15.38%)	0 (0%)	2 (15.38%)	8 (61.54%)
SHG-based images with AI models provide reliable fibrosis quantification	0 (0%)	5 (38.46%)	5 (38.46%)	1 (7.69%)	0 (0%)	0 (0%)	0 (0%)	7 (53.85%)
AI models that provide continuous values/metrics in addition to ordinal scores are more helpful to pathologists	1 (7.69%)	8 (61.54%)	9 (69.23%)	2 (15.38%)	1 (7.69%)	0 (0%)	1 (7.69%)	1 (7.69%)

**Fig. 1:**
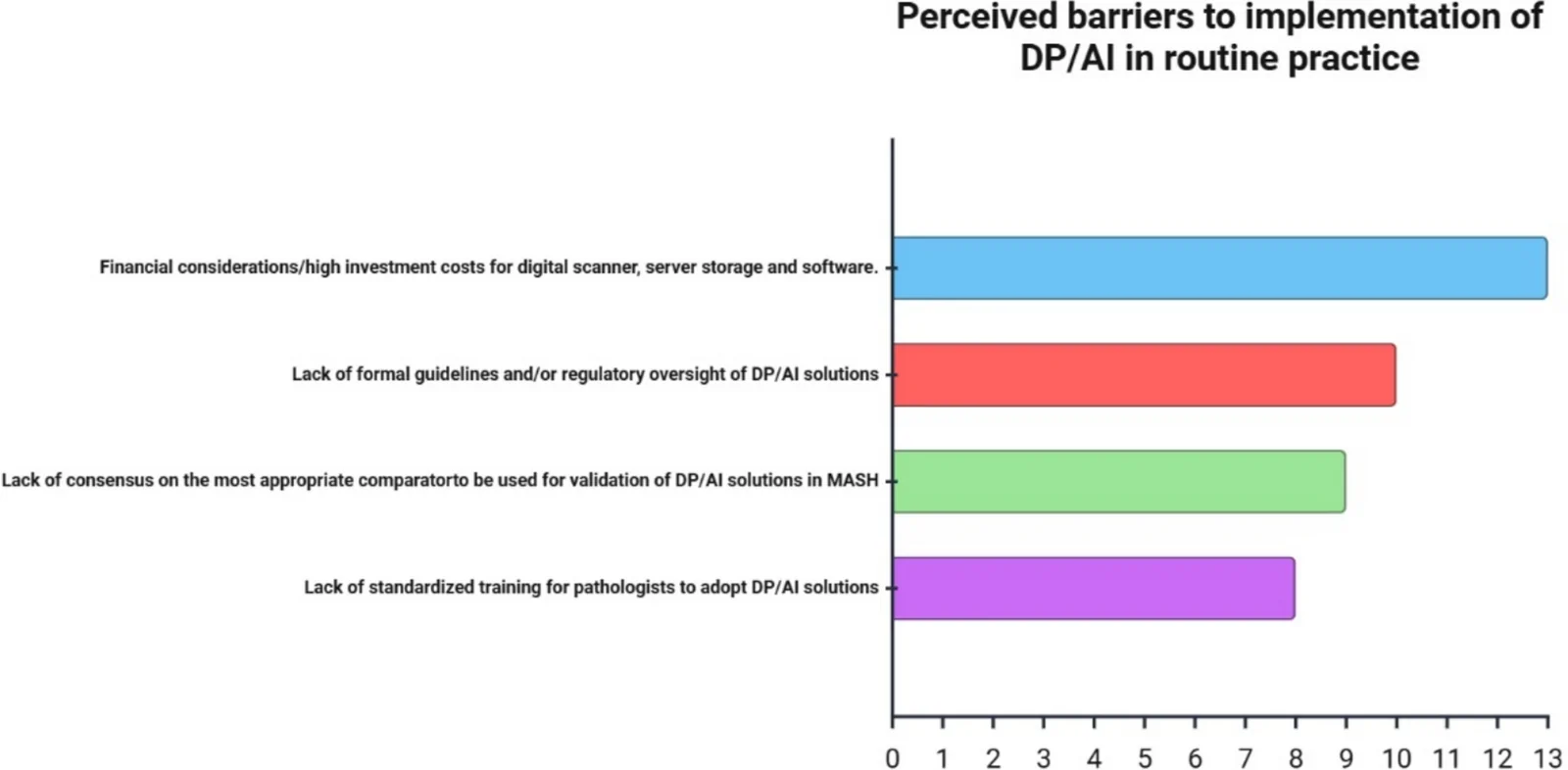
Liver pathologists’ perceived barriers to the implementation of digital pathology and AI into clinical workflows

The surveyed pathologists also highlighted some pertinent barriers to the successful implementation of digital pathology and AI into clinical and research workflows (Fig. 1).

Finally, all (*n* = 36) participants agreed on the need for defined standards for histological interpretation, data quality and expert pathologist input/oversight of AI models to facilitate their use in clinical practice or trials assessment.

## Discussion

This survey was designed to elucidate consensus around the potential of digital pathology and AI to augment liver histological interpretation in MAFLD/MASH and to identify barriers to adoption in clinical and research workflows in the Asia Pacific. The results provide important insights into current attitudes in the region around the promise and practicalities of AI-assisted digital pathology service adoption. The results broadly reflected a positive appraisal from both groups about the potential of AI-based digital pathology in augmenting MAFLD histological analyses. However, the survey also highlighted important considerations that warrant further discussion before a roadmap to consensus and large-scale adoption can be realized. It is important to acknowledge that a third of invited pathologists did not participate in the survey as opposed to only 8% of invited hepatologists. This resulted in a smaller than expected pool of liver pathologists and excessive weighting to individual responses that could have skewed the trend of responses, particularly as responses may reflect opinions of highly specialized or institutionally well-resourced experts rather than the broader regional practice landscape.

Conventional liver histological interpretations are technically challenging and suffer inherent limitations arising from ordinal scoring systems that lack the sensitivity necessary to capture nuanced therapeutic responses, morphological interpretations such as degree of ballooning and steatosis that are difficult to quantify and subject to interobserver disagreement, and slide quality control discrepancies. The majority of surveyed hepatologists concurred with this assessment around the limitations of current reporting methodology. However, pathologists had mixed opinions on the performance of conventional liver biopsy reporting, a discrepancy that could be explained by specialty-specific emphasis on interrelated yet distinct outcomes. Pathologists are adept at histological diagnosis and grading while hepatologists are more focused on how these pathological insights ultimately translate into clinical outcomes or applications which may require more granular tissue data signatures. AI-assisted biopsy analysis and quantitative histologic tools can provide nuanced outputs such as reporting the degree of fibrosis on a continuous scale to overcome the sensitivity problems stemming from ordinal reporting standards. However, these technologies require defined standards for data quality and validation by expert liver pathologists who ultimately will provide the oversight necessary for these tools to function optimally, a notion that both hepatologists and pathologists supported.

**Fig. 2:**
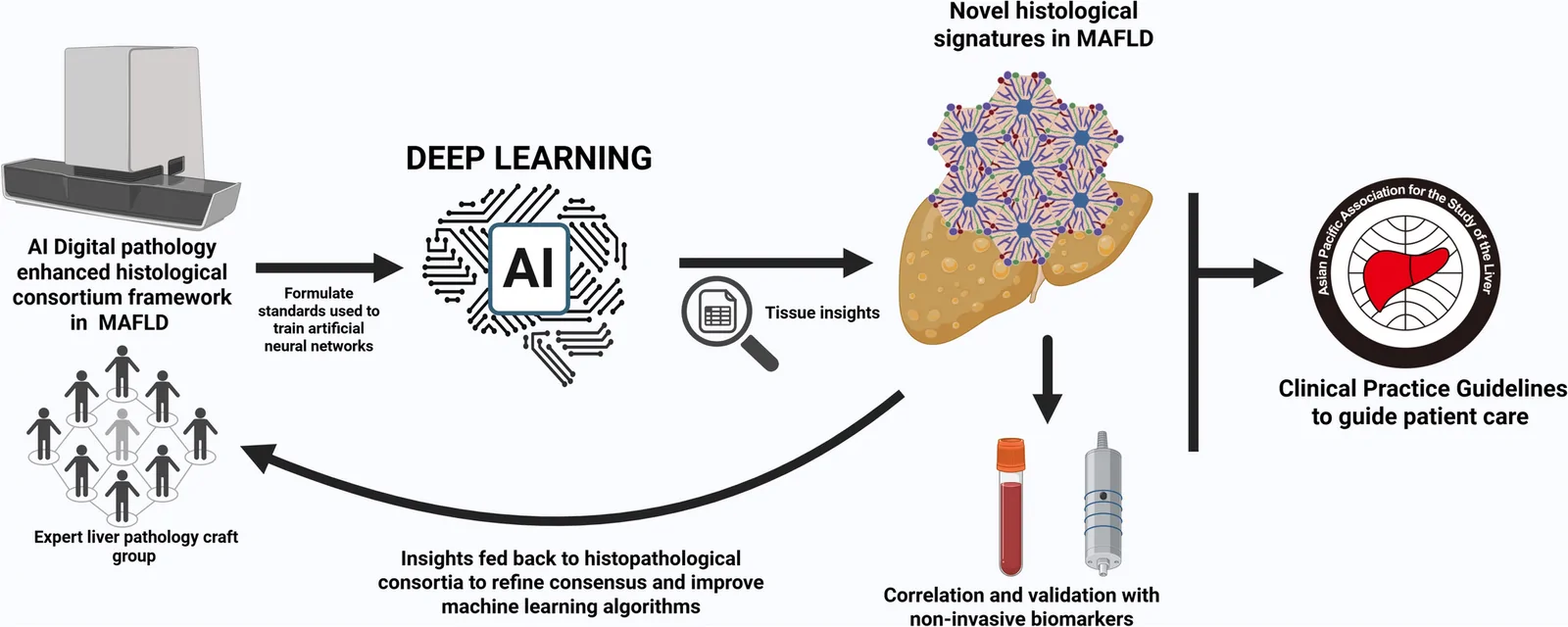
A proposed framework to drive advances in AI pathology assisted histological interpretation in MAFLD/MASH. Created with Biorender.com

Given the importance of liver fibrosis as the primary determinant of clinical outcomes, an important first step could be to formulate a hybrid system that augments existing ordinal fibrosis staging with clinically validated SHG/TPEF microscopy and AI-derived continuous fibrosis assessments (e.g., qFibrosis®) to redress efficacy endpoints in MAFLD/MASH clinical trials. Refinements to precisely define hepatocellular distortion (i.e., degree of ballooning, steatosis, etc.) will also be helpful. In this regard, initiatives such as the recently published global expert pathologist consensus designed to standardize the interpretation of cellular morphology, grade and stage of steatotic liver disease set an important precedent [5]. Moreover, developments such as the AIM-MASH system [6] which augmented the abilities of expert liver pathologists in assessing inflammation and MASH resolution are also welcome innovations in constructing clear frameworks for sensitive and reproducible histological reporting. Correlation of AI-assisted liver pathology analyses with clinical endpoints will then be the necessary validation for these platforms to be adopted in MASH clinical trials’ endpoint assessment. These data could potentially be retrospectively analyzed from the current phase 3 clinical trials that are underway. Data mining of biochemical and radiological signatures from trial datasets can also be calibrated against histological results to enhance the development of NITs which from a population policy perspective, are required to fulfill the bulk of clinical assessment of liver disease severity in a cost and risk effective manner (Fig. 2).

A significant number of pathologists were not routinely acquainted with quantitative fibrosis or AI capabilities in digital pathology, reflecting the significant cost and logistical barriers in accessing these technologies outside of research settings. This is a pertinent observation in the Asia Pacific region where there are wide differences in socio-economic development, health systems, and research footprints. It could also explain the observed cautious enthusiasm from the surveyed pathologists to incorporate augmented image analysis into existing workflows. Nevertheless, there is clear interest from both groups in the potential of the new technologies to refine liver diagnostics. Consequently, advocating to democratize access to these modalities should be a priority for apex regional bodies such as the APASL in conjunction with leading medical institutions, trials consortia, industry partners and governance bodies. There needs to be investment into updating training curricula in the region to include dedicated attention to digital pathology. Educational fora at specialty congresses (such as the APASL annual meetings) may also prove helpful. Such initiatives can serve to promulgate interdisciplinary collaboration, expand familiarity with the available technologies, standardize the operational nomenclature and enhance advocacy to achieve consensus around adoption in the region.

## Conclusion

AI and digital pathology are providing novel insights into liver histology and promise to herald a new quantitative era in the pathological sciences. These advances have significant benefits to be had in the MAFLD/MASH space where sensitivity and interobserver variability from current grading systems are suboptimal for trial analyses. As the survey identifies, there are important challenges to adoption but this effort sets the stage for pathologists and hepatologists in the Asia Pacific to spearhead initiatives to redefine pathological assessment and enhance clinical practice.

## Supplementary Information

Below is the link to the electronic supplementary material.

Supplementary file1 (DOCX 26 KB)

Supplementary file2 (DOCX 33 KB)

Supplementary file3 (XLSX 32 KB)

## Data Availability

All survey responses are available in de-identified format in Supplement 2.

## References

[1] Filozof C, Chow SC, Dimick-Santos L, et al. Clinical endpoints and adaptive clinical trials in precirrhotic nonalcoholic steatohepatitis: facilitating development approaches for an emerging epidemic. Hepatol Commun. 2017;1(7):577–585. 10.1002/hep4.1079 29404480 10.1002/hep4.1079 PMC5721443

[2] Naoumov NV, Brees D, Loeffler J, et al. Digital pathology with artificial intelligence analyses provides greater insights into treatment-induced fibrosis regression in NASH. J Hepatol. 2022;77(5):1399–1409. 10.1016/j.jhep.2022.06.018 35779659 10.1016/j.jhep.2022.06.018

[3] Kendall TJ, Chng E, Ren Y, Tai D, Ho G, Fallowfield JA. Outcome prediction in metabolic dysfunction-associated steatotic liver disease using stain-free digital pathological assessment. Liver Int. 2024;44(10):2511–2516. 10.1111/liv.16062 39109545 10.1111/liv.16062

[4] Eslam M, Fan J-G, Yu M-L, et al. The Asian Pacific Association for the Study of the Liver clinical practice guidelines for the diagnosis and management of metabolic dysfunction-associated fatty liver disease. Hepatol Int. 2025;19(2):261–301. 10.1007/s12072-024-10774-3 40016576 10.1007/s12072-024-10774-3

[5] Lackner C, Gouw ASH, Alves V, et al. Consensus position statements for the standardized application of histological grading and staging systems in MASH clinical trials. J Hepatol. 2025. 10.1016/j.jhep.2025.09.019 41072805 10.1016/j.jhep.2025.09.019

[6] Pulaski H, Harrison SA, Mehta SS, et al. Clinical validation of an AI-based pathology tool for scoring of metabolic dysfunction-associated steatohepatitis. Nat Med. 2025;31(1):315–322. 10.1038/s41591-024-03301-2 39496972 10.1038/s41591-024-03301-2 PMC11750710

