# Correction: The Asian Pacific Association of the Study of the Liver expert survey on artificial intelligence-assisted reporting of liver histopathology in metabolic dysfunction associated fatty liver disease

**DOI:** 10.1007/s12072-026-11131-2

**Published:** 2026-08-24

**Authors:** H. Elangovan, K. Akbary, A. Rastogi, A. Wee, G. Soon, L. Adams, E. Carr-Boyd, A. Clouston, C. L. Cooper, W. K. Chan, Y. Y. Dan, R. Dela-Cruz, G. Goh, S. S. Hamid, D. Q. Huang, T. Kawaguchi, H. Kim, W. Kim, S. U. Kim, J. D. Jia, C. J. Liu, F. Liu, W. Q. Leow, M. D. Muthiah, I. Ng, D. Payawal, A. F. Pan, S. Pervez, G. Shiha, T. Tanwandee, Y. Tanaka, L. Thiyaphat, M. Vij, Y. Yilmaz, F. Yilmaz, M. L. Yu, K. Zalata, M. H. Zheng, J. G. Fan, S. K. Sarin, V. Wong, J. George

**Affiliations:** 1 Storr Liver Centre, The Westmead Institute for Medical Research, Westmead Hospital, The University of Sydney, Sydney, NSW Australia; 2 HistoIndex Pte Ltd, Singapore, Singapore; 3 Department of Pathology, Institute of Liver and Biliary Sciences, New Delhi, India; 4 Department of Pathology, National University Hospital, Singapore, Singapore; 5 Department of Anatomical Pathology, Singapore General Hospital, Singapore, Singapore; 6 Medical School, University of Western Australia, Perth, Washington, Australia; 7 Histopathology Department, Auckland City Hospital, Auckland, New Zealand; 8 Centre for Liver Disease Research, School of Medicine (Southern), University of Queensland, Princess Alexandra Hospital, Ipswich, Australia; 9 Pathology Queensland, Princess Alexandra Hospital, Woolloongabba, Qld Australia; 10 Gastroenterology and Hepatology Unit, Department of Medicine, Faculty of Medicine, University of Malaya, Kuala Lumpur, Malaysia; 11 Division of Gastroenterology and Hepatology, Department of Medicine, National University Hospital, Singapore, Singapore; 12 The Medical City, Ortigas, Pasig City, Metro Manila, Philippines; 13 Department of Gastroenterology and Hepatology, Singapore General Hospital, Singapore, Singapore; 14 Department of Medicine, Aga Khan University, Karachi, Pakistan; 15 Division of Gastroenterology, Department of Medicine, Kurume University School of Medicine, Kurume, Japan; 16 Department of Pathology, Seoul National University Hospital, Seoul National University College of Medicine, Seoul, Republic of Korea; 17 Division of Gastroenterology and Hepatology, Department of Internal Medicine, Seoul Metropolitan Government Boramae Medical Center, Seoul National University College of Medicine, Seoul, Republic of Korea; 18 Department of Internal Medicine, Yonsei University College of Medicine, Seoul, South Korea; 19 Liver Research Center, Beijing Friendship Hospital, Capital Medical University, Beijing, 100050 China; 20 Division of Gastroenterology and Hepatology, Department of Internal Medicine, National Taiwan University Hospital, Taipei, Taiwan; 21 Peking University People’s Hospital, Peking University Hepatology Institute, Infectious Disease and Hepatology Center, Peking University People’s Hospital, Beijing, China; 22 Department of Pathology and State Key Laboratory of Liver Research, The University of Hong Kong, Pok Fu Lam, Hong Kong; 23 Department of Internal Medicine, Fatima University Medical Center, Valenzuela, Philippines; 24 Department of Pathology, Queen Elizabeth Hospital, Kota Kinabalu, Sabah, Malaysia; 25 Department of Pathology, Aga Khan University, Karachi, Pakistan; 26 Gastroenterology and Hepatology Unit, Internal Medicine Department, Faculty of Medicine, Mansoura University, Mansoura, Egypt; 27 Division of Gastroenterology, Department of Medicine, Faculty of Medicine Siriraj Hospital, Bangkok, Thailand; 28 Department of Gastroenterology and Hepatology, Kumamoto University, Kumamoto, Japan; 29 Division of Pathology, Chulabhorn International College of Medicine, Thammasat University, Pathum Thani, Thailand; 30 Department of Pathology, Dr Rela Institute and Medical Center, Chennai, Tamil Nadu India; 31 Department of Gastroenterology, School of Medicine, Recep Tayyip Erdoğan University, Rize, Türkiye; 32 Faculty of Medicine, Department of Pathology, Ege University, Izmir, Türkiye; 33 Hepatobiliary Division, Department of Internal Medicine, Center of Hepatitis Research, College of Medicine and Center of Metabolic Disorders and Obesity, Kaohsiung Medical University Hospital, Kaohsiung Medical University, Kaohsiung, Taiwan; 34 Anatomic Pathology Department, Faculty of Medicine, Mansoura University, Mansoura, Egypt; 35 Department of Hepatology, MAFLD Research Center, The First Affiliated Hospital of Wenzhou Medical University, Wenzhou, China; 36 Center for Fatty Liver, Department of Gastroenterology, Shanghai Key Lab of Pediatric Gastroenterology and Nutrition, Xinhua Hospital Affiliated to Shanghai Jiao Tong University School of Medicine, Shanghai, 200092 China; 37 Department of Hepatology, Institute of Liver and Biliary Sciences, New Delhi, India; 38 Department of Medicine and Therapeutics, Faculty of Medicine, The Chinese University of Hong Kong, Sha Tin, Hong Kong

**Correction: Hepatology International (2026) 20:539–549** **https://doi.org/10.1007/s12072-026-11092-6**

In the original version of this article, co-author Haeryoung Kim was inadvertently omitted from the author group. The author list should read: H. Elangovan · K. Akbary · A. Rastogi · A. Wee · G. Soon · L. Adams · E. Carr‑Boyd · A. Clouston · C. L. Cooper · W. K. Chan · Y. Y. Dan · R. Dela‑Cruz · G. Goh · S. S. Hamid · D. Q. Huang · T. Kawaguchi · H. Kim · W. Kim · S. U. Kim · J. D. Jia · C. J. Liu · F. Liu · W. Q. Leow · M. D. Muthiah · I. Ng · D. Payawal · A. F. Pan · S. Pervez · G. Shiha · T. Tanwandee · Y. Tanaka · L. Thiyaphat · M. Vij · Y. Yilmaz · F. Yilmaz · M. L. Yu · K. Zalata · M. H. Zheng · J. G. Fan · S. K. Sarin · V. Wong · J. George.

The affiliation details for Haeryoung Kim are: Department of Pathology, Seoul National University Hospital, Seoul National University College of Medicine, Seoul, Republic of Korea

The original article has been corrected.

