# Supplementary material for: The Asian Pacific Association of the Study of the Liver expert survey on artificial intelligence-assisted reporting of liver histopathology in metabolic dysfunction associated fatty liver disease
Source: Hepatol Int. 2026 May 13;20(3):539–49. doi: 10.1007/s12072-026-11092-6 (PMC13332892; doi:10.1007/s12072-026-11092-6)
Supplement: Supplementary file 1 — Supplementary file1 (DOCX 26 KB) [file 12072_2026_11092_MOESM1_ESM.docx]

**USE OF AI-BASED DIGITAL PATHOLOGY (DP/AI) IN MAFLD/MASH**

**Terminology**

DP = digital pathology based whole slide imaging

DP/AI = AI-based digital pathology

1. Please indicate your professional background:
   - Hepatologist or gastroenterologist
   - Pathologist (expert liver pathologist - evaluating >100 liver biopsies per year)
   - Pathologist (general pathologist with interest in liver pathology)
   - Pathologist (general pathologist)
2. How many years of experience do you have in your field?

- <5 years
- 5–10 years
- 11–20 years
- >20 years

**Statements**

Please rate your level of agreement using the following scale:
1 = Strongly Disagree
2 = Disagree
3 = Neither agree nor disagree
4 = Agree
5 = Strongly Agree

6 = Do not know/Prefer not to answer

**SECTION 1: QUESTIONS FOR BOTH HEPATOLOGISTS AND PATHOLOGISTS**

1. Liver histology is an imperfect gold standard for the evaluation of MASH and for the grading and staging of histological features of MAFLD.

☐ 1 ☐ 2 ☐ 3 ☐ 4 ☐ 5 ☐ 6

1. Liver histology is an imperfect gold standard for the development of non-invasive tests for MASH diagnosis and prognosis.

☐ 1 ☐ 2 ☐ 3 ☐ 4 ☐ 5 ☐ 6

1. Which scoring system do you use for research / clinical trials of MAFLD/MASH? (Select all that apply)

☐ NASH Clinical Research Network (NASH-CRN) scoring system

☐ Steatosis, Activity and Fibrosis (SAF) scoring system

☐ Other systems (please specify): _______________________________________

☐ No histological scoring

1. Which scoring system do you use in clinical practice for MAFLD/MASH? (Select all that apply)

☐ NASH Clinical Research Network (NASH-CRN) scoring system

☐ Steatosis, Activity and Fibrosis (SAF) scoring system

☐ Other systems (please specify): _______________________________________

☐ No histological scoring

1. Restricted ordinal histological categories of fibrosis staging are suboptimal for assessing fibrosis progression/regression in clinical trials.

☐ 1 ☐ 2 ☐ 3 ☐ 4 ☐ 5 ☐ 6

1. There is a need to integrate Digital Pathology with image analysis and/or artificial intelligence-based analysis with existing histological evaluation for fibrosis staging in the evaluation of MASH.

☐ 1 ☐ 2 ☐ 3 ☐ 4 ☐ 5 ☐ 6

1. There is a need to integrate Digital Pathology with image analysis and/or artificial intelligence-based analysis with existing histological evaluation for grading of steatosis in the evaluation of MASH.

☐ 1 ☐ 2 ☐ 3 ☐ 4 ☐ 5 ☐ 6

1. There is a need to integrate Digital Pathology with image analysis and/or artificial intelligence-based analysis with existing histological evaluation for grading of ballooning in the evaluation of MASH.

☐ 1 ☐ 2 ☐ 3 ☐ 4 ☐ 5 ☐ 6

1. There is a need to integrate Digital Pathology with image analysis and/or artificial intelligence-based analysis with existing histological evaluation for grading of lobular inflammation in the evaluation of MASH.

☐ 1 ☐ 2 ☐ 3 ☐ 4 ☐ 5 ☐ 6

1. DP/AI can improve the reproducibility of fibrosis scoring in MASH.

☐ 1 ☐ 2 ☐ 3 ☐ 4 ☐ 5 ☐ 6

1. DP/AI can improve the reproducibility of steatosis, lobular inflammation, and ballooning scoring in MASH.

☐ 1 ☐ 2 ☐ 3 ☐ 4 ☐ 5 ☐ 6

1. DP/AI can reduce subjectivity in interpreting borderline MASH grading and fibrosis stages.

☐ 1 ☐ 2 ☐ 3 ☐ 4 ☐ 5 ☐ 6

1. DP/AI can be an effective decision support tool for pathologists in grading and staging MASH.

☐ 1 ☐ 2 ☐ 3 ☐ 4 ☐ 5 ☐ 6

1. Which histological trial endpoints could DP/AI most effectively support or enhance? (Select all that apply)

☐ ≥1-stage fibrosis regression

☐ No worsening of steatohepatitis

☐ Quantitative change in collagen proportionate area

☐ Fibrosis progression prevention

☐ Composite histological scores (e.g., NAS + fibrosis)

☐ Not suitable for endpoint use at this stage

1. DP/AI should supplement, not replace, human pathology review in clinical practice.

☐ 1 ☐ 2 ☐ 3 ☐ 4 ☐ 5 ☐ 6

1. DP/AI based fibrosis evaluation can serve as the comparator for development of non-invasive tests.

☐ 1 ☐ 2 ☐ 3 ☐ 4 ☐ 5 ☐ 6

1. Development, validation and standardization of DP/AI tools should be an area of high priority in assessing MAFLD/MASH, particularly for clinical trials and drug development.

☐ 1 ☐ 2 ☐ 3 ☐ 4 ☐ 5 ☐ 6

1. Formal Professional Society guidelines (e.g. APASL, EASL, AASLD) should be established for the use of DP/AI in assessing MAFLD.

☐ 1 ☐ 2 ☐ 3 ☐ 4 ☐ 5 ☐ 6

1. Defined standards for data quality, expert input, validation, performance and transparency of AI models should be formulated for their use in clinical practice or trials for MAFLD assessment.

☐ 1 ☐ 2 ☐ 3 ☐ 4 ☐ 5 ☐ 6

1. Validation of AI algorithms for MASH/fibrosis scoring should be multi-centre across different population cohorts to ensure reproducibility and generalizability.

☐ 1 ☐ 2 ☐ 3 ☐ 4 ☐ 5 ☐ 6

1. Pathologists should retain final authority in grading and fibrosis staging, even when AI tools are used.

☐ 1 ☐ 2 ☐ 3 ☐ 4 ☐ 5 ☐ 6

1. AI model performance should be reported together with documentation of pre-analytical parameters (e.g. specimen handling, fixation, section thickness, staining, scanner specifications) to ensure result interpretability and reproducibility.

☐ 1 ☐ 2 ☐ 3 ☐ 4 ☐ 5 ☐ 6

1. The following validation metrics should be prioritized for DP/AI based MASH/fibrosis assessment tools (select all that apply):

☐ Accuracy

☐ Reproducibility

☐ Correlation with clinical outcomes

☐ Interpretability (explainability)

☐ Generalizability across populations

1. What do you consider the most appropriate primary comparator when validating AI tools for the assessment of MASH? (Select all that apply)

- Manual histopathology by expert liver pathologist
- Elastography (e.g., FibroScan, MRE)
- Composite non-invasive scoring systems (e.g., FIB-4, NAFLD fibrosis score)
- Clinical outcomes (e.g., liver-related events, mortality)
- Central histopathology review by expert panel
- Composite trial endpoints (e.g., ≥1-stage fibrosis regression + no worsening of steatohepatitis)
- Concordance with regulatory agency-approved scoring systems (e.g., NASH CRN)
- Other (please specify): ___________

**SECTION 2: QUESTIONS FOR PATHOLOGISTS**

1. Have you used or developed DP/AI tools for liver pathology or fibrosis assessment?

- Yes
- No

1. Are you currently using digital pathology (e.g., whole slide imaging) in your liver assessments?

- Yes (routinely)
- Yes (for research only)
- No

1. Existing conventional ordinal histological staging systems are semi-quantitative and categorical without fibrosis quantification (collagen amount) to reflect intra-stage changes; hence, they are not ideal/optimum for assessing progression/regression of fibrosis in MASH.

☐ 1 ☐ 2 ☐ 3 ☐ 4 ☐ 5 ☐ 6

1. Semi-quantitative scores of ballooning degeneration of hepatocytes and lobular inflammation are suboptimal for application in clinical trials due to non-standardized and divergent definitions.

☐ 1 ☐ 2 ☐ 3 ☐ 4 ☐ 5 ☐ 6

1. There is limited inter-pathologist agreement for grading of hepatocyte ballooning.

☐ 1 ☐ 2 ☐ 3 ☐ 4 ☐ 5 ☐ 6

1. A 1-point grade difference in hepatocyte ballooning, for NAS improvement in clinical trials, is difficult to record using the existing histological scoring system.

☐ 1 ☐ 2 ☐ 3 ☐ 4 ☐ 5 ☐ 6

1. Hepatocyte ballooning score 0 required for MASH resolution in clinical trials is difficult to assess and is associated with marked subjectivity.

☐ 1 ☐ 2 ☐ 3 ☐ 4 ☐ 5 ☐ 6

1. There is limited inter-pathologist agreement for grading of lobular inflammation.

☐ 1 ☐ 2 ☐ 3 ☐ 4 ☐ 5 ☐ 6

1. A 1-point grade difference in lobular inflammation, for NAS improvement in clinical trials, is difficult to record using the existing histological scoring system.

☐ 1 ☐ 2 ☐ 3 ☐ 4 ☐ 5 ☐ 6

1. DP/AI can assist pathologists in assessments of MASH.

☐ 1 ☐ 2 ☐ 3 ☐ 4 ☐ 5 ☐ 6

1. Digital pathology for review by pathologists or AI platforms that use stained images should include digitized images of glass slides stained with hematoxylin and eosin, and a matrix stain.

☐ 1 ☐ 2 ☐ 3 ☐ 4 ☐ 5 ☐ 6

1. Digitized whole slide images are as reliable as glass slides for assessing fibrosis in MASH.

☐ 1 ☐ 2 ☐ 3 ☐ 4 ☐ 5 ☐ 6

1. Digitized whole slide images are as reliable as glass slides for assessing steatosis in MASH.

☐ 1 ☐ 2 ☐ 3 ☐ 4 ☐ 5 ☐ 6

1. Digitized whole slide images are as reliable as glass slides for assessing hepatocyte ballooning in MASH.

☐ 1 ☐ 2 ☐ 3 ☐ 4 ☐ 5 ☐ 6

1. Digitized whole slide images are as reliable as glass slides for assessing lobular inflammation in MASH.

☐ 1 ☐ 2 ☐ 3 ☐ 4 ☐ 5 ☐ 6

1. Have you used DP/AI on unstained sections using Second Harmonic Generation (SHG)-based imaging

☐ Yes

☐ No

1. DP/AI on unstained sections, including Second Harmonic Generation (SHG)-based imaging, is advantageous to DP/AI on stained sections due to circumvention of potential pre-analytical errors (e.g., staining variability).

☐ 1 ☐ 2 ☐ 3 ☐ 4 ☐ 5 ☐ 6

1. SHG-based images have better resolution and contrast than routine histochemical stains for fibrosis assessment.

☐ 1 ☐ 2 ☐ 3 ☐ 4 ☐ 5 ☐ 6

1. SHG-based images alone without AI solutions can assist pathologists in MASH biopsy evaluation, especially for fibrosis assessment.

☐ 1 ☐ 2 ☐ 3 ☐ 4 ☐ 5 ☐ 6

1. SHG-based images with AI models provide reliable fibrosis quantification.

☐ 1 ☐ 2 ☐ 3 ☐ 4 ☐ 5 ☐ 6

1. AI models that provide continuous values/metrics in addition to ordinal scores are more helpful to pathologists.

☐ 1 ☐ 2 ☐ 3 ☐ 4 ☐ 5 ☐ 6

1. Which of the following is/are major hurdles for the implementation of DP/AI? (Select all that apply)

☐ Financial considerations/high investment costs for digital scanner, server storage and software.

☐ Lack of formal guidelines and/or regulatory oversight of DP/AI solutions

☐ Lack of consensus on the most appropriate comparator or “ground truth” to be used for validation of DP/AI solutions in MASH for clinical practice and clinical trials

☐ Lack of standardized training for pathologists to adopt DP/AI solutions in routine practice

1. Digital pathology training should be incorporated into Pathology residency and fellowship programmes.

☐ 1 ☐ 2 ☐ 3 ☐ 4 ☐ 5 ☐ 6

1. Pathologists should receive formal training on the use of the specific DP/AI model(s) before deployment in routine clinical practice and/or clinical trials.

☐ 1 ☐ 2 ☐ 3 ☐ 4 ☐ 5 ☐ 6
