# Supplementary material for: The Asian Pacific Association of the Study of the Liver expert survey on artificial intelligence-assisted reporting of liver histopathology in metabolic dysfunction associated fatty liver disease
Source: Hepatol Int. 2026 May 13;20(3):539–49. doi: 10.1007/s12072-026-11092-6 (PMC13332892; doi:10.1007/s12072-026-11092-6)
Supplement: Supplementary file 2 — Supplementary file2 (DOCX 33 KB) [file 12072_2026_11092_MOESM2_ESM.docx]

|  | **Strongly Agree** | **Agree** | **Overall Agree** | **Neither Agree nor Disagree** | **Disagree** | **Strongly Disagree** | **Overall Disagree** | **Do not know/prefer not to answer** |
| --- | --- | --- | --- | --- | --- | --- | --- | --- |
| Liver histology is an imperfect gold standard for the evaluation of MASH and for the grading and staging of histological features of MAFLD | 10 (43.48%) | 10 (43.48%) | 20 (86.96%) | 0 (0%) | 2 (8.7%) | 1 (4.35%) | 3 (13.04%) | 0 (0%) |
| Liver histology is an imperfect gold standard for the development of non-invasive tests for MASH diagnosis and prognosis | 9 (39.13%) | 11 (47.83%) | 20 (86.96%) | 0 (0%) | 3 (13.04%) | 0 (0%) | 3 (13.04%) | 0 (0%) |
| Restricted ordinal histological categories of fibrosis staging are suboptimal for assessing fibrosis progression/regression in clinical trials | 12 (52.17%) | 9 (39.13%) | 21 (91.3%) | 1 (4.35%) | 0 (0%) | 1 (4.35%) | 1 (4.35%) | 0 (0%) |
| There is a need to integrate Digital Pathology with image analysis and/or artificial intelligence-based analysis with existing histological evaluation for fibrosis staging in the evaluation of MASH | 14 (60.87%) | 8 (34.78%) | 22 (95.65%) | 0 (0%) | 0 (0%) | 0 (0%) | 0 (0%) | 1 (4.35%) |
| There is a need to integrate Digital Pathology with image analysis and/or artificial intelligence-based analysis with existing histological evaluation for grading of steatosis in the evaluation of MASH | 12 (52.17%) | 7 (30.43%) | 19 (82.61%) | 1 (4.35%) | 2 (8.7%) | 0 (0%) | 2 (8.7%) | 1 (4.35%) |
| There is a need to integrate Digital Pathology with image analysis and/or artificial intelligence-based analysis with existing histological evaluation for grading of ballooning in the evaluation of MASH | 14 (60.87%) | 8 (34.78%) | 22 (95.65%) | 0 (0%) | 0 (0%) | 0 (0%) | 0 (0%) | 1 (4.35%) |
| There is a need to integrate Digital Pathology with image analysis and/or artificial intelligence-based analysis with existing histological evaluation for grading of lobular inflammation in the evaluation of MASH | 12 (52.17%) | 10 (43.48%) | 22 (95.65%) | 0 (0%) | 0 (0%) | 0 (0%) | 0 (0%) | 1 (4.35%) |
| DP/AI can improve the reproducibility of fibrosis scoring in MASH | 14 (60.87%) | 8 (34.78%) | 22 (95.65%) | 0 (0%) | 0 (0%) | 0 (0%) | 0 (0%) | 1 (4.35%) |
| DP/AI can improve the reproducibility of steatosis, lobular inflammation, and ballooning scoring in MASH | 12 (52.17%) | 9 (39.13%) | 21 (91.3%) | 1 (4.35%) | 0 (0%) | 0 (0%) | 0 (0%) | 1 (4.35%) |
| DP/AI can reduce subjectivity in interpreting borderline MASH grading and fibrosis stages | 10 (43.48%) | 12 (52.17%) | 22 (95.65%) | 0 (0%) | 0 (0%) | 0 (0%) | 0 (0%) | 1 (4.35%) |
| DP/AI can be an effective decision support tool for pathologists in grading and staging MASH | 8 (34.78%) | 14 (60.87%) | 22 (95.65%) | 0 (0%) | 0 (0%) | 0 (0%) | 0 (0%) | 1 (4.35%) |
| DP/AI should supplement, not replace, human pathology review in clinical practice | 11 (47.83%) | 9 (39.13%) | 20 (86.96%) | 3 (13.04%) | 0 (0%) | 0 (0%) | 0 (0%) | 0 (0%) |
| DP/AI based fibrosis evaluation can serve as the comparator for development of non-invasive tests | 8 (34.78%) | 11 (47.83%) | 19 (82.61%) | 2 (8.7%) | 1 (4.35%) | 0 (0%) | 1 (4.35%) | 1 (4.35%) |
| Development, validation and standardization of DP/AI tools should be an area of high priority in assessing MAFLD/MASH, particularly for clinical trials and drug development | 12 (52.17%) | 11 (47.83%) | 23 (100%) | 0 (0%) | 0 (0%) | 0 (0%) | 0 (0%) | 0 (0%) |

Hepatologists’ response to the questionnaire

Hepatologist response to Questionnaire

|  | **Strongly Agree**  Pathologists’ response to the questionnaire | **Agree** | **Overall Agree** | **Neither Agree nor Disagree** | **Disagree** | **Strongly Disagree** | **Overall Disagree** | **Do not know/prefer not to answer** |
| --- | --- | --- | --- | --- | --- | --- | --- | --- |
| Liver histology is an imperfect gold standard for the evaluation of MASH and for the grading and staging of histological features of MAFLD | 3 (23.08%) | 5 (38.46%) | 8 (61.54%) | 1 (7.69%) | 4 (30.77%) | 0 (0%) | 4 (30.77%) | 0 (0%) |
| Liver histology is an imperfect gold standard for the development of non-invasive tests for MASH diagnosis and prognosis | 3 (23.08%) | 3 (23.08%) | 6 (46.15%) | 1 (7.69%) | 4 (30.77%) | 1 (7.69%) | 5 (38.46%) | 1 (7.69%) |
| Restricted ordinal histological categories of fibrosis staging are suboptimal for assessing fibrosis progression/regression in clinical trials | 0 (0%) | 4 (30.77%) | 4 (30.77%) | 5 (38.46%) | 4 (30.77%) | 0 (0%) | 4 (30.77%) | 0 (0%) |
| DP/AI can improve the reproducibility of fibrosis scoring in MASH | 3 (23.08%) | 8 (61.54%) | 11 (84.62%) | 2 (15.38%) | 0 (0%) | 0 (0%) | 0 (0%) | 0 (0%) |
| DP/AI can improve the reproducibility of steatosis, lobular inflammation, and ballooning scoring in MASH | 2 (15.38%) | 8 (61.54%) | 10 (76.92%) | 1 (7.69%) | 2 (15.38%) | 0 (0%) | 2 (15.38%) | 0 (0%) |
| DP/AI can reduce subjectivity in interpreting borderline MASH grading and fibrosis stages | 2 (15.38%) | 9 (69.23%) | 11 (84.62%) | 0 (0%) | 2 (15.38%) | 0 (0%) | 2 (15.38%) | 0 (0%) |
| DP/AI can be an effective decision support tool for pathologists in grading and staging MASH | 2 (15.38%) | 10 (76.92%) | 12 (92.31%) | 1 (7.69%) | 0 (0%) | 0 (0%) | 0 (0%) | 0 (0%) |
| DP/AI should supplement, not replace, human pathology review in clinical practice | 8 (61.54%) | 4 (30.77%) | 12 (92.31%) | 0 (0%) | 0 (0%) | 1 (7.69%) | 1 (7.69%) | 0 (0%) |
| DP/AI based fibrosis evaluation can serve as the comparator for development of non-invasive tests | 2 (15.38%) | 9 (69.23%) | 11 (84.62%) | 2 (15.38%) | 0 (0%) | 0 (0%) | 0 (0%) | 0 (0%) |
| Development, validation and standardization of DP/AI tools should be an area of high priority in assessing MAFLD/MASH, particularly for clinical trials and drug development | 3 (23.08%) | 9 (69.23%) | 12 (92.31%) | 1 (7.69%) | 0 (0%) | 0 (0%) | 0 (0%) | 0 (0%) |
| DP/AI on unstained sections, including Second Harmonic Generation (SHG)-based imaging, is advantageous to DP/AI on stained sections due to circumvention of potential pre-analytical errors (e.g., staining variability) | 0 (0%) | 1 (7.69%) | 1 (7.69%) | 5 (38.46%) | 0 (0%) | 0 (0%) | 0 (0%) | 7 (53.85%) |
| SHG-based images have better resolution and contrast than routine histochemical stains for fibrosis assessment | 0 (0%) | 2 (15.38%) | 2 (15.38%) | 3 (23.08%) | 0 (0%) | 0 (0%) | 0 (0%) | 8 (61.54%) |
| SHG-based images alone without AI solutions can assist pathologists in MASH biopsy evaluation, especially for fibrosis assessment | 0 (0%) | 2 (15.38%) | 2 (15.38%) | 1 (7.69%) | 2 (15.38%) | 0 (0%) | 2 (15.38%) | 8 (61.54%) |
| SHG-based images with AI models provide reliable fibrosis quantification | 0 (0%) | 5 (38.46%) | 5 (38.46%) | 1 (7.69%) | 0 (0%) | 0 (0%) | 0 (0%) | 7 (53.85%) |
| AI models that provide continuous values/metrics in addition to ordinal scores are more helpful to pathologists | 1 (7.69%) | 8 (61.54%) | 9 (69.23%) | 2 (15.38%) | 1 (7.69%) | 0 (0%) | 1 (7.69%) | 1 (7.69%) |
| There is a need to integrate Digital Pathology with image analysis and/or artificial intelligence-based analysis with existing histological evaluation for fibrosis staging in the evaluation of MASH | 1 (7.69%) | 8 (61.54%) | 9 (69.23%) | 3 (23.08%) | 1 (7.69%) | 0 (0%) | 1 (7.69%) | 0 (0%) |
| There is a need to integrate Digital Pathology with image analysis and/or artificial intelligence-based analysis with existing histological evaluation for grading of steatosis in the evaluation of MASH | 1 (7.69%) | 8 (61.54%) | 9 (69.23%) | 3 (23.08%) | 1 (7.69%) | 0 (0%) | 1 (7.69%) | 0 (0%) |
| There is a need to integrate Digital Pathology with image analysis and/or artificial intelligence-based analysis with existing histological evaluation for grading of ballooning in the evaluation of MASH | 1 (7.69%) | 7 (53.85%) | 8 (61.54%) | 3 (23.08%) | 2 (15.38%) | 0 (0%) | 2 (15.38%) | 0 (0%) |
| There is a need to integrate Digital Pathology with image analysis and/or artificial intelligence-based analysis with existing histological evaluation for grading of lobular inflammation in the evaluation of MASH | 0 (0%) | 10 (76.92%) | 10 (76.92%) | 2 (15.38%) | 1 (7.69%) | 0 (0%) | 1 (7.69%) | 0 (0%) |

Pathologists’ response to the questionnaire
